# Oligomeric State of β-Coronavirus Non-Structural Protein 10 Stimulators Studied by Small Angle X-ray Scattering

**DOI:** 10.3390/ijms241713649

**Published:** 2023-09-04

**Authors:** Wolfgang Knecht, S. Zoë Fisher, Jiaqi Lou, Céleste Sele, Shumeng Ma, Anna Andersson Rasmussen, Nikos Pinotsis, Frank Kozielski

**Affiliations:** 1 Department of Biology & Lund Protein Production Platform & Protein Production Sweden, Lund University, Sölvegatan 35, 22362 Lund, Sweden; wolfgang.knecht@biol.lu.se (W.K.); zoe.fisher@biol.lu.se (S.Z.F.); celeste.sele@biol.lu.se (C.S.); anna.andersson_rasmussen@biol.lu.se (A.A.R.); 2 European Spallation Source ERIC, P.O. Box 176, 22100 Lund, Sweden; 3 School of Pharmacy, University College London, 29-39 Brunswick Square, London WC1N 1AX, UK; jiaqi.lou.13@ucl.ac.uk (J.L.); shumeng.ma.20@ucl.ac.uk (S.M.); 4 Institute of Structural and Molecular Biology, Birkbeck College, London WC1E 7HX, UK

**Keywords:** SARS-CoV-2, COVID-19, non-structural proteins, nsp10, SAXS, oligomeric state, conformational changes

## Abstract

The β-coronavirus family, encompassing Severe Acute Respiratory Syndrome Coronavirus 2 (SARS-CoV-2), Severe Acute Respiratory Syndrome Coronavirus (SARS), and Middle East Respiratory Syndrome Coronavirus (MERS), has triggered pandemics within the last two decades. With the possibility of future pandemics, studying the coronavirus family members is necessary to improve knowledge and treatment. These viruses possess 16 non-structural proteins, many of which play crucial roles in viral replication and in other vital functions. One such vital protein is non-structural protein 10 (nsp10), acting as a pivotal stimulator of nsp14 and nsp16, thereby influencing RNA proofreading and viral RNA cap formation. Studying nsp10 of pathogenic coronaviruses is central to unraveling its multifunctional roles. Our study involves the biochemical and biophysical characterisation of full-length nsp10 from MERS, SARS and SARS-CoV-2. To elucidate their oligomeric state, we employed a combination of Multi-detection Size exclusion chromatography (Multi-detection SEC) with multi-angle static light scattering (MALS) and small angle X-ray scattering (SAXS) techniques. Our findings reveal that full-length nsp10s primarily exist as monomers in solution, while truncated versions tend to oligomerise. SAXS experiments reveal a globular shape for nsp10, a trait conserved in all three coronaviruses, although MERS nsp10, diverges most from SARS and SARS-CoV-2 nsp10s. In summary, unbound nsp10 proteins from SARS, MERS, and SARS-CoV-2 exhibit a globular and predominantly monomeric state in solution.

## 1. Introduction

Coronaviruses (CoVs) belong to the RNA viruses in the *Coronaviridae* family, order *Nidovirales*. Within this family, there are four genera, which are α, β, γ, and σ coronaviruses. Among these, the β-coronaviruses are known to infect humans, with seven of them having been identified as affecting the human host. While four of these viruses typically cause mild symptoms similar to the common cold, the remaining three have led to deadly outbreaks over the past two decades [1]. One of the pathogenic CoVs, SARS, caused a limited outbreak in China in 2002, resulting in approximately 8000 infections and a mortality rate close to 10% [2,3]. A decade later, another CoV outbreak occurred in Saudi Arabia, named MERS, which had a lower infection rate (ca. 2500) but a significantly higher mortality rate at ~35% [4]. 

The most recent CoV outbreak emerged at the end of 2019, caused by a novel CoV later named SARS-CoV-2, leading to the COVID-19 pandemic. SARS-CoV-2 has caused a global ongoing pandemic, affecting the entire world population with over 670 million infections and more than 6.7 million deaths, although unofficial estimates suggest higher numbers [5]. It is now widely accepted that SARS-CoV-2 is becoming endemic in many countries [6]. Given the regular recurrence of CoVs over the past two decades, it is essential to study and understand how these viruses infect humans, replicate in the human host, and develop strategies to handle infections to reduce severe disease and mortality. 

SARS-CoV-2 is a single-stranded positive-sense RNA virus, with a genome that is approximately 30 kb in size, coding for three distinct classes of proteins [7]. The virion is composed of four structural proteins named spike, envelope, nucleocapsid, and membrane proteins. Additionally, seven accessory proteins, known for their contribution to viral pathogenicity [8], may vary significantly between different β-CoVs [9]. However, the largest class of proteins comprises the 16 non-structural proteins (nsp1 to nsp16), many of which play vital roles in viral replication [10] as part of the replication-transcription complex (RTC).

Some of these nsps are already validated drug targets, such as Paxlovid (Nirmatrelvir and Ritonavir), which targets the main protease (nsp5) [11,12,13], and Remdesivir, inhibiting the RNA-dependent RNA polymerase (RdRp) complex (nsp12-nsp7 and nsp8) [14]. As their application is limited and the development of resistance is widely anticipated, the search for new drugs targeting other potential protein targets becomes crucial [15,16]. Recently, clinical trials have shown that approved monoclonal antibodies lose their efficacy against omicron variants, leading to their approval being reversed [17,18], indicating that the pandemic is far from over. 

Several other non-structural proteins are considered potential targets due to their essential roles in viral replication such as the helicase (nsp13) [19], bifunctional nsp14 containing 3′-to-5′ exoribonuclease (ExoN) and N7 methyltransferase (N7-MTase) activities [20], and nsp16 with 2′-O-methyltransferase activity (2′-O-MTase) [10,21]. However, many other non-structural proteins are also being investigated for their potential as targets.

Among these nsps, one of the central players is nsp10, a scaffolding protein that interacts with and stimulates at least two other non-structural proteins, nsp14 and nsp16 [22,23]. Nsp10 stimulates the ExoN activity of SARS and SARS-CoV-2 nsp14, but not its N7-MTase activity [24] by forming a 1:1 complex [25]. Similarly, it also stimulates the 2′-O-MTase activity of nsp16, forming a heterodimeric complex reported for all three pathogenic β-CoVs [21,26,27] (Protein Data Bank (PDB) example entries 2XYR and 5YN8). This makes nsp10 vital not only in the RNA cap formation process [28] but also for RNA proofreading [29]. Recently, it has even been proposed that nsp14, nsp10, and nsp16 can form a heterotrimeric complex, with nsp14 and nsp16 overlapping on nsp10 in a particular N-terminal region of nsp14, termed the “lid”, showing high structural flexibility [30,31]. Successfully targeting nsp10 could lead to the inhibition of ExoN and N7-MTase functions, thus preventing RNA cap formation and hindering RNA proofreading by the ExoN domain, which corrects falsely incorporated bases by the proofreading RdRp complex [32]. However, several knowledge gaps remain, such as the oligomeric state of the unbound form of nsp10 in solution compared to its state in forming various heterodimeric complexes. 

**Figure 1:**
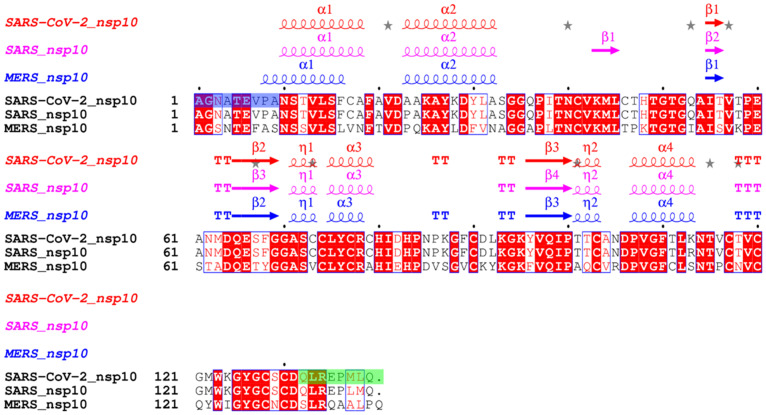
Protein sequence and structural alignment of full-length nsp10 proteins from SARS-CoV-2, SARS and MERS. Identical residues are shown in white on a red background, similar residues are shaded in red and distinct residues are displayed in black. Truncated residues of the long SARS-CoV-2 nsp10 construct are highlighted in green, and additional missing residues of the short SARS-CoV-2 nsp10 construct are highlighted in blue. The percent identity between SARS-CoV-2 and SARS nsp10 proteins is 97.1%. In contrast, the percent identity between SARS-CoV-2 and MERS nsp10 proteins is 59.4%. The alignment was carried out using CLUSTAL OMEGA, 1.2.4 [34] while the secondary structure was assigned by DSSP as implemented in ESPript 3.0 [35].

**Figure 2:**
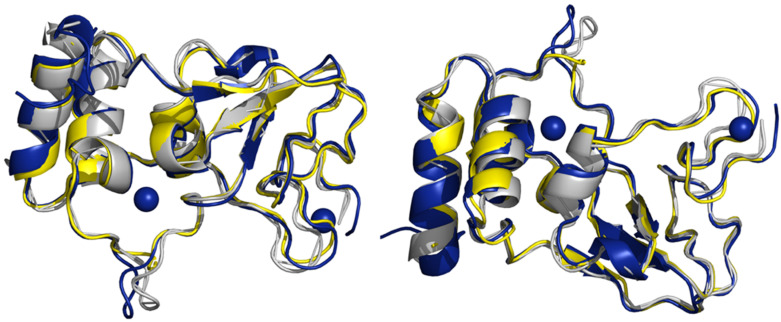
Overlay of crystal structures of nsp10 from MERS, SARS, and SARS-CoV-2. Ribbon diagram of crystal structures of nsp10 MERS (grey, PDB entry 5YN5), SARS (yellow, PDB entry 2G9T), and SARS-CoV-2 (blue, PDB entry 7DIY) with structural zinc atoms shown as blue spheres. View on right is rotated by 180° along the horizontal axis with respect to view on the left. The three nsp10 crystal structures downloaded from the RCSB PDB were superposed in Pymol (v2.4.1) [36].

In this project, we aim to characterise and compare the oligomeric state of nsp10 proteins from SARS, MERS, and SARS-CoV-2. The sequence alignment in Figure 1 illustrates the high degree of nsp10 sequence conservation (59.4%) in MERS compared to both SARS and SARS-COV-2, while nsp10 is 97.1% conserved between SARS and SARS-CoV-2. Figure 2 depicts a structural overlay of nsp10 crystal structures obtained from SARS, MERS, and SARS-CoV-2, revealing a remarkable degree of structural homology (r.m.s.d. is <1 Å over all atoms). Our comprehensive analysis, employing various techniques, consistently indicates that these nsp10s predominantly exists as monomers in solution. To gain further insight into their shapes, we conducted Small Angle X-ray Scattering (SAXS) experiments [33], confirming a conserved shape for nsp10s from SARS, MERS, and SARS-CoV-2, consistent with the known nsp10 crystal structures. 

## 2. Results and Discussion

**Figure 3:**
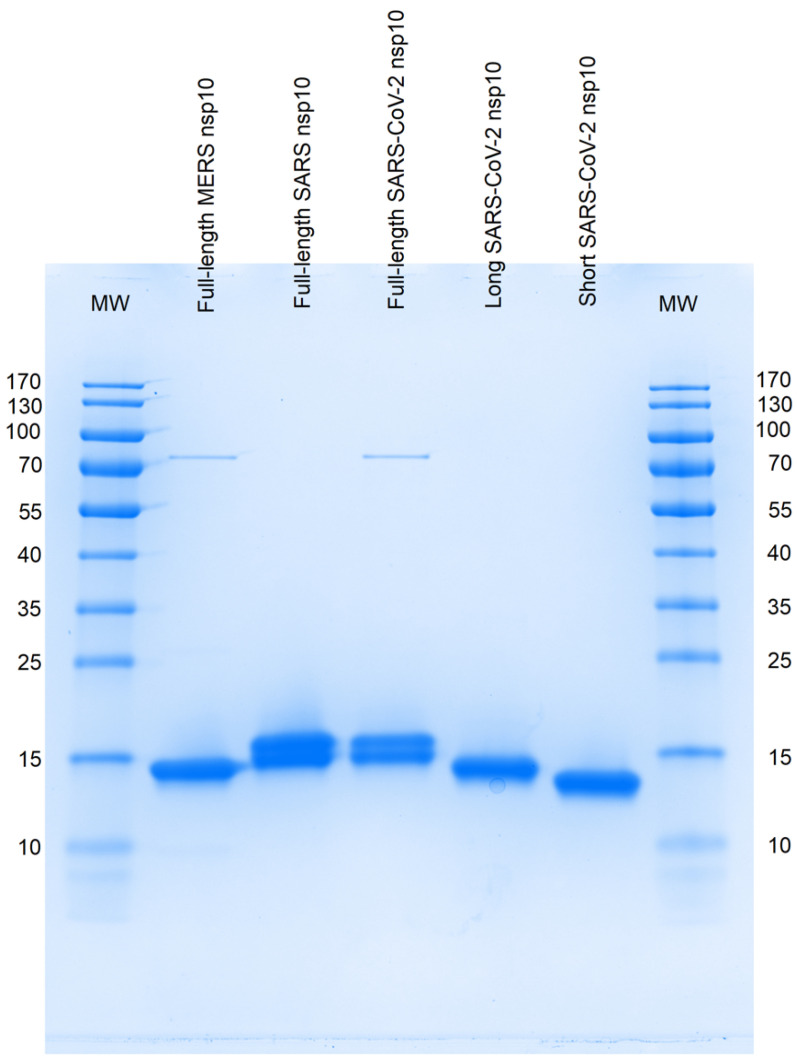
SDS-PAGE of SARS-CoV-2, SARS and MERS nsp10 constructs purified for this study. Lane designation: MW contains the molecular weight marker proteins (in kDa) and the nsp10 proteins in the other lanes are indicated by names. The faint bands slightly above 70 kDa were identified by mass-spectrometry analysis as chaperone protein DNAK from *E. coli* (UniProtKB ID: P0A6Y8; calculated MW: 69.1 kDa).

We prepared nsp10 constructs of varying lengths for MERS, SARS, and SARS-CoV-2, including full-length proteins for all three CoVs, alongside two shorter constructs for SARS-CoV-2. The purification process yielded an estimated purity of >95% for all proteins (Figure 3). On the SDS-PAGE, we observed that full-length MERS nsp10 migrates close to the lower band of each full-length SARS and SARS-CoV-2 nsp10. However, the splitting of the protein bands, evident in lanes C and D by SDS-PAGE, was not observed when examining the proteins in solution by multi-detection SEC (Table 1). We, therefore, conducted high-resolution mass spectrometry (HRMS) using all five protein constructs to validate the protein species. HRMS measurements show single species corresponding to the expected masses of all nsp10 constructs, suggesting that the double-bands and the lower migrating band for MERS nsp10 are technique-related artefacts of SDS-PAGE (Figure S1). Additionally, we detected a minor band at approximately 75 kDa in full-length MERS (lane B) and SARS-CoV-2 (lane D) nsp10, later identified as the *E. coli* chaperone DNAK (UniProtKB P0A6Y8, calculated MW of 69.1 kDa) through mass spectrometry analysis of the excised SDS-PAGE gel. 

To determine the oligomeric state nsp10s from SARS, MERS, and SARS-CoV-2, we conducted Multi-detection SEC experiments, combining SEC separation, light scattering at two different angles, and differential viscometer measurements. The use of Multi-detection SEC allowed us to make reliable and highly sensitive measurements of the absolute molecular weight and particle distribution in solution. The Multi-detection SEC results revealed that the molecular weight of all three full-length nsp10s in solution did not significantly differ. Furthermore, we found that all constructs, especially the full-length nsp10 proteins, predominantly exist as monomers in solution (Table 1). Figure S2 shows representative Multi-detection SEC traces.

Notably, the full-length MERS nsp10 demonstrated exceptional monodispersity, whereas full-length SARS and SARS-CoV-2 nsp10s formed a minor pool of dimers (2 to 3%) in solution. The shorter, “artificial” SARS-CoV-2 protein constructs exhibited a more substantial extent of dimer (3–11%) and trimer (up to 3%) formation in solution. However, it is important to emphasise that across all three β-CoV nsp10s, the predominant state in solution was monomeric. Figure S3, derived from SEC-MALS experiments [37] with the three SARS-CoV-2 nsp10 constructs, further confirmed the predominantly monomeric nature of nsp10, with only slight variations in the mass fraction. These findings reinforce the reliability and significance of our earlier Multi-detection SEC data, providing valuable insights into the oligomeric states of nsp10s from various coronaviruses. 

The SARS nsp10 crystal structure was previously reported as monomeric, with one molecule in the asymmetric unit. However, SEC analysis revealed a dimeric state in solution [38]. It is important to note that the SEC analysis utilised a truncated SARS nsp10 construct, not the full-length protein, as we did in our study. In other work, a fusion between SARS nsp10 and nsp11 has been reported to crystallise as a dodecamer [39]. Nevertheless, there is no supporting evidence for the existence of such a nsp10-nsp11 fusion protein. In contrast, our novel findings clearly demonstrate that full-length nsp10s from various medically relevant β-CoVs predominantly exist as monomers, whereas truncated nsp10 constructs have a tendency to oligomerise into dimers and trimers, albeit to a very low extent. In conclusion, our study confirms that the β-CoV nsp10s studied here primarily form monomers in solution, which aligns with their functional roles as stimulators of nsp14 and nsp16 activities. These nsp10s act in a 1:1 complex with their binding partners or participate as one partner in a nsp10-nsp14-nsp16 heterotrimeric complex.

To gain further structural insights into the various nsp10 proteins in solution, we characterised the three SARS-CoV-2 nsp10 constructs (short, long, full-length) along with the full-length SARS and MERS nsp10 proteins. In-line SEC was performed just before measuring SAXS data (SEC-SAXS). Protein concentrations were adjusted to 10.0 mg/mL, except for the short SARS-CoV-2 nsp10 construct (8.0 mg/mL) and MERS nsp10 (7.3 mg/mL). All scattering intensity plots displayed a similar profile to what was observed in SEC-MALS, showing a major peak with an almost negligent peak at an earlier elution volume (Figure S4).

**Figure 4:**
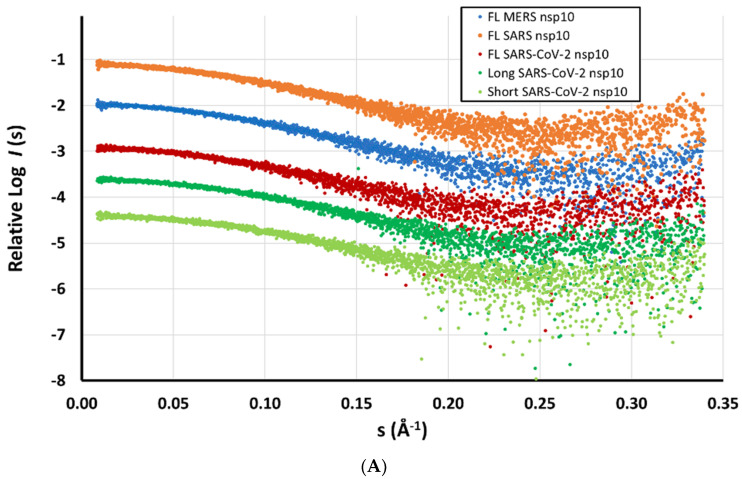

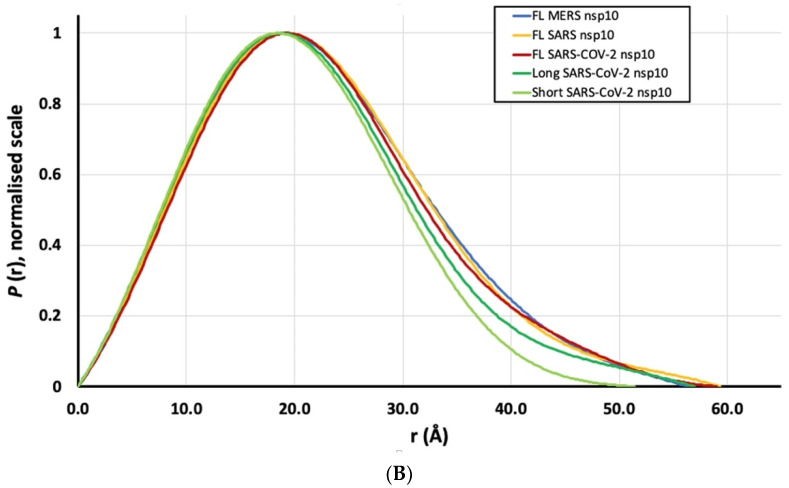
(**A**) Experimental SAXS data measured for nsp10 proteins from SARS-CoV-2 (short, long and full-length) and the full-length nsp10 proteins from MERS and SARS coloured in light green, dark green, red, blue and yellow respectively in this figure and Figure 5. Data are shown in relative scales. (**B**) Distance distribution profiles of the scattering data from (**A**).

The Radius of Gyration (R_g_) was consistent for all full-length nsp10s at approximately 17.2–17.3 Å, slightly smaller for the shorter SARS-CoV-2 nsp10 constructs. Similar values were calculated for the maximum distance (D_max_), ranging from 51 to 59 Å, as well as for the estimated molecular masses. All statistics are summarised in Table S2. Overall, SAXS revealed a similar shape of a globular domain for all full-length and shorter nsp10s (Figure 4). The distance distribution layout of the two shorter nsp10 proteins of SARS-CoV-2 closely resembled a Gaussian layout, suggesting a more spherical shape (Figure 4B). Kratky plots suggested a rigid and identical shape for all measured samples (Figure S5).

**Figure 5:**
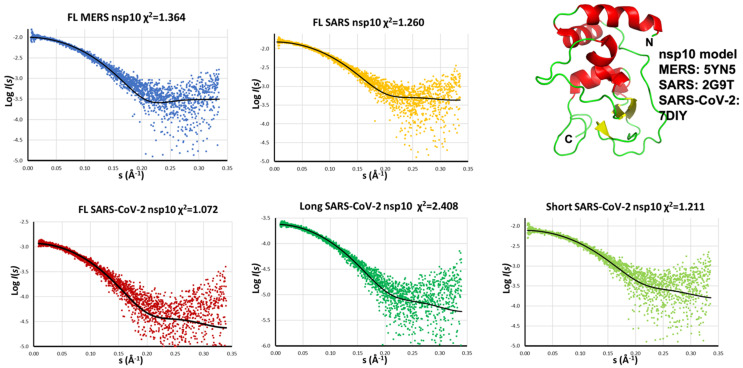
Comparison between the measured scattering data and the calculated scattering curve from the model (in black and in cartoon representation on the right). Discrepancy χ^2^ is shown on the header of each plot. The nsp10 coordinates were extracted from the PDB.

We further compared the scattering data for all five samples to reference coordinates. For SARS-CoV-2 nsp10, the coordinates in complex with the nsp14-ExoN domain were trimmed to include residues 6-128, representing the most characteristic of the domain fold. As for the other two viral nsp10s, we used the coordinates as found in the PDB. The discrepancy χ^2^, calculated from CRYSOL, ranged from 1.072 up to 1.364 for the four measured proteins and 2.408 for the long nsp10 SARS-CoV-2 construct. These values indicated and excellent agreement between the solution scattering data and the crystal structure of nsp10 (Figure 5).

**Figure 6:**
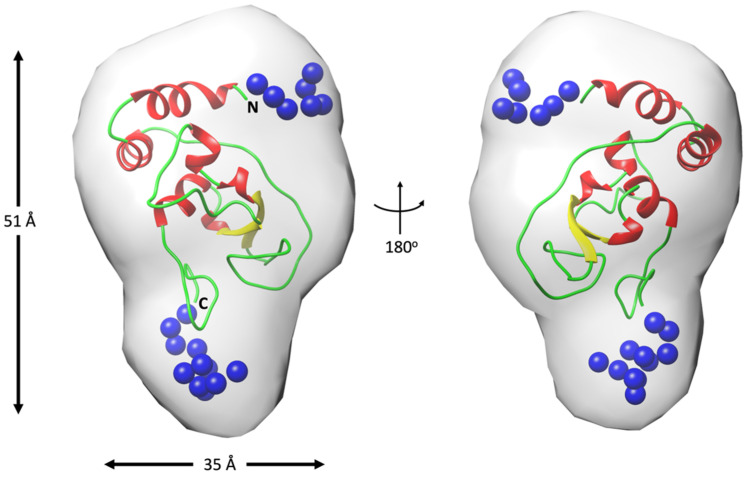
Superimposition of the full-length ab initio nsp10 SARS-CoV-2 model shown as a molecular envelope with the rigid body/ab initio model of the same protein shown as cartoon in two different orientations. The ab initio envelope was generated in chimera using a map covering the calculated coordinates with a resolution of 13 Å. The extra residues added in the rigid body/ab initio are shown as blue spheres. Maximum dimensions for two of the orientations are indicated on the left as well as the N- and C-terminal boundaries of the model.

To gain a deeper understanding of the roles played by the residues at the N- and C-terminal regions of nsp10, we generated a low-resolution model for full-length SARS-CoV-2 nsp10. Our approach involved two main steps: firstly, we generated 20 ab initio models using the program Dammif [40]; then, aligning these models, we used their average envelope as the starting search volume for a slow mode ab initio calculation using the software Dammin from ATSAS suite (v3.2.1) [41]. The final model converged with a discrepancy χ^2^ against the raw data of 0.9876, signifying a very reasonable fit although slightly overfit. Moreover, we independently applied a rigid body/ab initio approach to determine the positions of the missing residues at both termini, utilising the software BUNCH from ATSAS suite (v3.2.1) [42]. The final model reported a discrepancy χ^2^ of 1.01897 against the raw data, indicating a similar and highly accurate fit compared to the ab initio model. Notably, upon superposition, these two models exhibited a reasonable fit, effectively demonstrating that the termini significantly contributed to the compact core of the structure (Figure 6). This result is in agreement with the observations from the Kratky plots, which convincingly suggest that nsp10 forms a rigid and stable protein domain. Encouragingly, very similar ab initio/rigid body-refined models were obtained for full-length SARS and MERS nsp10 proteins. 

Overall, all nsp10 proteins from SARS-CoV-2, SARS, and MERS exhibit an almost identical low-resolution shape of a monomeric domain. While this is expected for the SARS-CoV-2 and SARS nsp10 proteins, where the sequence identity is 97.1%, it is less obvious for MERS nsp10, which is only 59.4% identical to SARS-Cov-2 nsp10. Furthermore, the two full-length SARS-CoV-2 nsp10 models (ab initio and rigid body), combined with the evaluation of the data by CRYSOL using a SARS-CoV-2 nsp10 crystal structure, strongly suggest that extra residues at the termini of all proteins do not affect the overall fold and shape of the proteins.

In conclusion, full-length nsp10 stimulators from medically relevant β-coronaviruses, such as SARS, MERS, and SARS-CoV2, are predominantly compact monomers in solution, as revealed by SAXS. This finding is consistent with the crystal structures of SARS and SARS-CoV-2 nsp10, which also exist as monomers in their unbound forms, in the absence of their nsp14 and nsp16 binding partners. An exception is the nsp10-nsp11 fusion protein, which has been shown to crystallise as a dodecamer. Truncated SARS-CoV-2 nsp10 constructs display a higher propensity to oligomerise and may even form trimers, explaining why a truncated SARS nsp10 construct has been reported as a dimer using size exclusion chromatography. Our work represents the first integration of results on various β-coronavirus nsp10s into a single model for nsp10 structure and function.

## 3. Materials and Methods

### 3.1. Subcloning, Expression and Purification of β-CoV nsp10 Proteins

Expression constructs for nsp10 from SARS-CoV-2 (UniProtKB: P0DTD1), SARS (UniProtKB: P0C6X7) and MERS (UniProtKB: K9N7C7) were synthesised at Genscript and subcloned into *E. coli* expression vector ppSUMO-2. Due to the cloning strategy, they contain up to two nonspecific residues (TM) at the N-terminus of the proteins. Expressed proteins contain an N-terminal, cleavable His-SUMO double tag aiding in the purification of the various proteins. The features of the expression clones are summarised in Table S1. A short SARS-CoV-2 nsp10 construct was designed as previously described [43], covering residues Asn4264 (renumbered to Asn10 for convenience) to Gln4385, with a total length of 123 residues. This construct lacks residues at both the N- and C-termini. A long SARS-CoV-2 nsp10 construct consists of 133 residues and includes the N-terminus of the protein but is still devoid of the C-terminus. Full-length SARS-CoV-2 nsp10 contains 139 residues. Full-length SARS nsp10 is a protein of 139 residues. The protein sequence of SARS nsp10 (NP_828868, 139 residues), located between Ala4231 to Gln4369 of pp1a/pp1ab was used to generate the expression clone. MERS full-length nsp10 contains 140 residues. 

The long and the short SARS-CoV-2 nsp10 proteins were expressed and purified as recently described [43]. Full-length SARS-CoV-2 nsp10 was expressed and purified as recently reported [44]. The full-length SARS and MERS nsp10s were purified using the same strategy employed for full-length SARS-Cov-2 nsp10. In brief, plasmids were transformed into Rosetta (DE3) competent cells (Merck KGaA, Darmstadt, Germany) and inoculated onto LB agar plates supplemented with 50 µg/mL kanamycin and 34 µg/mL chloramphenicol. For full-length MERS nsp10, the plasmid was transformed into BL21-Gold (DE3) competent cells (Agilent Technologies, Santa Clara, CA, USA) and inoculated onto an LB agar plate supplemented with 50 µg/mL kanamycin.

A single colony from each LB agar culture was inoculated into 30 mL TB medium supplemented with the same antibiotics as above and incubated at 37 °C and 220 RPM overnight; 5 mL of each culture was inoculated into each 1 L TB medium with the same antibiotics and incubated at 37 °C and 220 RPM until the OD_600_ reached 0.6–1.0. Cultures were cooled down to 18 °C and 1 mM IPTG was added to induce protein expression. The induced cultures were kept at 18 °C, and 220 RPM for 20–24 h. After expression, cultures were centrifuged at 8000× *g* at 4 °C for 20 min to remove the medium. Cell pellets were resuspended in nsp10 buffer A (50 mM sodium phosphate buffer (NaPO_4_) pH 8.0, 300 mM NaCl, 20 mM Imidazole and 1 mM PMSF), flash-frozen in liquid nitrogen and stored at −80 °C.

Cell pellets were thawed followed by sonication on ice for 10 cycles (30 s on, 60 s off) at a 16 kHz ultrasonic frequency, followed by centrifugation at 50,000× *g* at 4 °C for 1 h to remove cell debris. The supernatant was loaded into a 5 mL HisTrap FF crude column (Cytiva Life Sciences, Buckinghamshire, UK) pre-equilibrated with nsp10 buffer B (50 mM NaPO_4_ pH 8.0, 300 mM NaCl and 20 mM Imidazole). The loaded column was washed with 50 column volumes (CVs) nsp10 buffer B, followed by elution with 20 CVs nsp10 buffer C (50 mM NaPO_4_ pH 8.0, 300 mM NaCl and 250 mM Imidazole). Collected elution fractions were evaluated by SDS-PAGE and those containing nsp10 were pooled. ULP1 and 2 mM DTT were added to the pooled sample and dialysed against 2 L nsp10 dialysis buffer (50 mM NaPO_4_ pH 8.0, 300 mM NaCl, 20 mM Imidazole and 2 mM DTT) at 4 °C overnight to cleave off the SUMO and His double tag from nsp10. Cleaved nsp10 was purified through 2 × 5 mL HisTrap FF crude columns pre-equilibrated with nsp10 buffer B to remove any uncleaved fusion protein, His-tagged ULP1 protease and other contaminations. Buffer exchanges against final buffer (50 mM Tris-HCl pH 8.0 and 150 mM NaCl) were carried out by using 15 mL Amicon Ultra Centrifugal Units with 10,000 MWCO (Merck KGaA, Darmstadt, Germany).

### 3.2. MALDI-TOF/TOF Mass Spectrometry

SDS-PAGE gel bands were cut into 1 × 1 mm gel pieces and washed three times for 30 min by incubation with 400 µL 50% acetonitrile (ACN, Sigma-Aldrich, Dorset, UK) and 50 mM ammonium bicarbonate (ABC, Sigma-Aldrich). The gel pieces were dehydrated using 100% ACN before 25 µL of 12 ng/µL sequence grade modified trypsin porcine (Promega, Tokyo, Japan) in 25 mM ABC was added. The gel pieces in the digestion buffer were incubated on ice for 3 h before further incubation overnight at 37 °C. The following day Trifluoroacetic acid was added to a final concentration of 0.5% and peptides were extracted into a new tube ready for analysis by MALDI mass spectrometry.

MS and MS/MS spectra were acquired using an Autoflex Speed MALDI TOF/TOF mass spectrometer (Bruker Daltonics, Bremen, Germany) in positive reflector mode. Matrix solution, 0.5 µL consisting of 5 mg/ml α-cyano-4-hydroxy cinnamic acid, 80% acetonitrile, 0.1% TFA, was added to 1 µL peptide sample on a MALDI stainless steel plate. MS spectra were externally calibrated using Peptide Calibration Standard II (Bruker Daltonics, Billerica, MA, USA) containing nine standard peptides (Bradykinin Fragment 1–7 (757.40), Angiotensin II (1046.54), Angiotensin I, *m*/*z* 1296.68; Substance P, *m*/*z* 1347.74; Bombesin *m*/*z* 1619.82; Renin Substrate,1758.93; ACTH clip 1–17, *m*/*z* 2093.09; ACTH clip 18–39, *m*/*z* 2465.20; Somatostatin 28, *m*/*z* 3147.47).

The identification of proteins was based on both MS and MS/MS data and was carried out with the Mascot Daemon software (version 2.4). The following search settings were used: trypsin as protease, two allowed missed cleavage sites, 50 ppm MS accuracy for peptides and 0.2 Da for MS/MS fragments, variable modifications: Oxidation (M). The files were searched against all organisms in SwissProt (updated 221008).

### 3.3. High-Resolution Mass Spectrometry

High-resolution mass spectrometry measurements were performed on a 1290 Infinity II UHPLC system (Agilent Technologies, Santa Clara, CA, USA) connected to a Q-TOF LC–MS system (Agilent Technologies, 6545XT) equipped with a dual Agilent jet stream high sensitivity electrospray ionisation (ESI) source. LC and LC–MS data were acquired in MassHunter (Agilent Technologies). Data analysis was performed in MassHunter Qualitative Analysis with BioConfirm add-on (v.11.0).

One microlitre of each nsp10, dissolved in the HPLC-grade chromatography water at the concentration of 0.25 mg/ml, was injected into the system and went through a 50 × 2.1 mm, 5 µM, 1000 Å PLRP-S Reversed-Phase column (Agilent Technologies). The mobile phase consists of 95% water and 5% acetonitrile initially, gradually changing to 60% water and 40% acetonitrile at 9 min, then gradually shifting to 0% water and 100% acetonitrile at 10 min and held for 1 min, and finally gradually returning to 95% water and 5% acetonitrile at 12 min. The flow rate was set to 0.4 mL/min, and the column temperature was maintained at 24 °C. Operational parameters for the Q-TOF source were as follows: drying gas temperature: 350 °C, drying gas flow: 12 L/min, nebulizer pressure: 35 psig, sheath gas temperature: 350 °C, sheath gas flow: 11 L/min, nozzle voltage: 1000 V, capillary voltage: 4000 V, and fragmentor voltage at 200 V. The system was operated in Extended Dynamic Range (2 GHz) mode.

### 3.4. Multi-Detection SEC Experiments

The in-solution oligomeric state of the various nsp10 proteins was evaluated in running buffer composed of 50 mM Tris-HCl pH 8.0 and 150 mM NaCl; 100 µg of protein were injected in triplicate in a Multi-detection SEC system (Malvern Panalytical, Malvern, UK) composed of the OMNISEC RESOLVE module (integrating a Superdex 75 Increase 10/300 GL column (Cytiva, Uppsala, Sweden), with a combined pump, degasser, autosampler and column oven) and the OMNISEC REVEAL, an integrated multi-detector module (Right Angle Light Scattering (RALS) 90° angle and Low Angle Light Scattering (LALS) 7° angle), differential refractive index, viscometer and diode-array-based UV/Vis spectrometer). The flow rate used was 0.5 ml/min and the detectors were normalised with bovine serum albumin (Thermo Fisher Scientific, Waltham, MA, USA). The sample changer was cooled down to 4 °C while the column oven and detector compartment were temperature controlled at 25 °C. Data were collected and analysed with the Multi-detection SEC integrated software (v11.32) provided by Malvern. The protein concentration was determined using an average refractive index increment (dn/dc) of 0.185 mL/g. Based on the reproducibility of the results, one injection from the triplicate was chosen for the calculations.

### 3.5. SEC-MALS Experiments

The concentration for each protein was adjusted to 5 mg/mL. The samples were measured in an HPLC (Agilent 1100) in line connected to an eight-channel light scattering detector (Wyatt DAWN 8+) and a differential refractive index detector (Wyatt Optilab T-rex). The size-exclusion chromatography column (Superdex 75 10/300, Cytiva, Tokyo, Japan) was pre-equilibrated using the running buffer (10 mM HEPES pH 7.5, 300 mM NaCl, 1.5% (*v*/*v*) Glycerol and 2 mM DTT) for 2 CVs and then 100 μL of each protein sample were injected using the auto-injector module of the HPLC at a flow rate of 0.5 mL/min. The main peaks were analysed using the ASTRA v6 software (Wyatt).

### 3.6. Small Angle X-ray Scattering (SAXS)

After the initial characterisation of nsp10s in solution by Multi-detection SEC and SEC-MALS experiments, we used SAXS to study the overall shape and properties of various nsp10 proteins. The proteins were thawed on ice and injected into a size-exclusion chromatography column (Superdex 75 10/300, Cytiva, Tokyo, Japan) to remove aggregates and exchange buffers to the running buffer (10 mM HEPES pH 7.5, 300 mM NaCl, 1.5% (*v*/*v*) Glycerol and 2 mM DTT). The proteins were then concentrated using centrifugal filters (Amicon Ultra 10k, Sigma-Aldrich) and measured at synchrotron beamline B21 at Diamond Light Source, UK. Each sample was injected into the size-exclusion chromatography column (Superdex 75 10/300, Cytiva) linked to an HPLC system (Agilent Technologies 1200) and was subsequently measured in-line on the X-ray cell. Data were recorded on an Eiger detector under vacuum. The sample concentrations were adjusted to 10.0 mg/mL except for the short nsp10 protein construct measured at 8.0 mg/mL and full-length MERS nsp10 measured at 7.3 mg/mL; 50 μL of each sample was injected into the column. The scattering profiles for all samples indicated a clear separation between the major peak corresponding to the monomeric proteins and a small peak corresponding to an oligomeric form of the protein (Figure S4), similar to the SEC-MALS scattering profiles (Figure S3).

The data were processed using the program CHROMIXS [45] where baseline (buffer) and peak (protein) were interactively selected based on the intensity peak and the calculated radius of gyration (R_g_) values for each recorded point. All data were processed through the ATSAS suite (v3.2.1) [46] where the R_g_ (either from the Guinier approximation or the distance distribution function), the maximum particle dimension (D_max_), and the excluded volume (Porod) were evaluated using standard procedures. The molecular masses of the solutes were evaluated from the Porod volume and also from the ratio of forward scattering I(0) of each sample; bovine serum albumin was used as a reference. The scattering from the high-resolution models was evaluated by CRYSOL [47] while the ab initio modeling was calculated using DAMMIF and DAMMIN [40,41]. Rigid body modeling was performed by BUNCH [42]. Data collection statistics, parameters, and additional details of the SAXS measurements are summarised in Table S2.

For comparing SAXS data of nsp10 constructs to published crystal structures, we chose the SARS-CoV-2 nsp10 structure bound to nsp14 ExoN domain (PDB entry 7DIY) by removing nsp14 and restricting nsp10 to residues 6–128. This represents a conserved fold also found in the apo SARS-CoV2 nsp10 (PDB entry 6ZPE) but has a less ordered N-terminal region. In the structure of the SARS-CoV-2 nsp10 bound to nsp16, the N-terminal α-helix is disordered and at a different position compared to the two structures mentioned above. The high-resolution structure for the MERS nsp10 was derived from PDB entry 5YN5 (nsp10/nsp16 complex) and for SARS from PDB entry 2G9T.

## Figures and Tables

**Table 1:** Molecular weights and oligomerisation of various nsp10s proteins determined by multi-detection SEC. Multi-detection SEC runs with various nsp10 for the three β-CoVs. Only peaks with a relative amount of >1.0% are included in the table. MW and relative amount are average ± SD from three experiments.

Protein		Monomer		Dimer		Trimer	
	Calculated MW (Da)	MW (Da)	Relative Amount (%)	MW (Da)	Relative Amount (%)	MW (Da)	Relative Amount (%)
Short SARS-CoV-2 nsp10	13,272	13,954 ± 76	97.0 ± 1.9	27,741 ± 257	3.0 ± 1.9	n/a	n/a
Long SARS-CoV-2 nsp10	14,026	14,688 ± 70	85.0 ± 1.2	29,993 ± 253	11.0 ± 0.5	46,389 ± 1546	3.0 ± 0.9
Full-length SARS-CoV-2 nsp10	15,022	15,595 ± 131	93.6 ± 2.3	32,090 ± 837	3.0 ± 0.2	n/a	n/a
Full-length SARS nsp10	15,075	15,678 ± 4	97.6 ± 0.4	29,730 ± 376	2.2 ± 0.1	n/a	n/a
Full-length MERS nsp10	15,122	15,615 ± 52	98.7 ± 0.4	n/a	n/a	n/a	n/a

## Data Availability

The SASBDB accession codes fo the five nsp10 proteins are as follows. SASDS69, full length SARS-CoV-2 nsp10. SASDS79, long SARS-CoV-2 nsp10, C-terminal truncation construct. SASDS89, short SARS-CoV-2 nsp10, truncated at N- and C-termini. SASDS99, full length SARS nsp10. SASDSA9, full length MERS nsp10.

## Supplementary Materials

The following supporting information can be downloaded at: https://www.mdpi.com/1422-0067/24/17/13649/s1.

## Abbreviations

ABC	Ammonium Bicarbonate
ACN	Acetonitrile
CoV	Coronavirus
COVID-19	Coronavirus Disease 2019
DTT	DL-Dithiothreitol
ExoN	3′-to-5′ exoribonuclease
HEPES	4-(2-hydroxyethyl)-1-piperazineethanesulfonic acid
HRMS	High-resolution Mass Spectrometry
IPTG	Isopropyl-β-D-thiogalactopyranoside
LALS	Low Angle Light Scattering
MALS	Multi Angle Light Scattering
MERS	Middle East Respiratory Syndrome
NaPO_4_	Sodium phosphate buffer
Nsp	non-structural protein
MTase	Methyltransferase
PDB	Protein Data Bank
PMSF	Phenylmethylsulfonylfluorid
RdRp	RNA-dependent RNA polymerase
RTC	Replication-transcription complex
RALS	Right Angle Light Scattering
SARS	Severe Acute Respiratory Syndrome
SARS-CoV-2	Severe Acute Respiratory Syndrome Coronavirus 2
SAXS	Small Angle X-ray Scattering
SEC	Size-Exclusion Chromatography
Tris	Tris(hydroxymethyl)aminomethane
ULP1	Ubiquitin-like-specific protease 1
