# Supplementary material for: Oligomeric State of β-Coronavirus Non-Structural Protein 10 Stimulators Studied by Small Angle X-ray Scattering
Source: Int J Mol Sci. 2023 Sep 4;24(17):13649. doi: 10.3390/ijms241713649 (PMC10563069; doi:10.3390/ijms241713649)
Supplement: Supplementary file 1 [file ijms-24-13649-s001.zip › ijms-2505179-supplementary.pdf]

Supplementary Information

Oligomeric state of β-coronavirus non-structural protein 10 stimulators studied by Small Angle X-ray Scattering

Wolfgang Knecht [1], Zoë Fisher [1,2], Jiaqi Lou [3], Céleste Sele [1], Shumeng Ma [3], Anna Andersson Rasmussen [1], Nikos Pinotsis [4] and Frank Kozielski [3]

[1] Department of Biology & Lund Protein Production Platform & Protein Production Sweden, Lund University, Sölvegatan 35, 22362 Lund, Sweden; wolfgang.knecht@biol.lu.se; celeste.sele@biol.lu.se; anna.andersson_rasmussen@biol.lu.se

[2]European Spallation Source ERIC, P.O. Box 176, 22100 Lund, Sweden; Zoe.Fisher@ess.eu

[3]School of Pharmacy, University College London, London, 29-39 Brunswick Square, London WC1N 1AX, United Kingdom; jiaqi.lou.13@ucl.ac.uk; shumeng.ma.20@ucl.ac.uk

[4]Institute of Structural and Molecular Biology, Birkbeck College, WC1E 7HX London, United Kingdom. n.pinotsis@mail.cryst.bbk.ac.uk

Correspondence: f.kozielski@ucl.ac.uk

Contents

**Table S1**	Summary of nsp10 constructs from β-CoVs	3
**Table S2**	SAXS data collection and scattering parameters	4
**Figure S1**	Denatured high-resolution mass spectra (HRMS)	6
**Figure S2**	Representative OmniSEC traces	11
**Figure S3**	SEC-MALS analysis of three SARS-CoV-2 nsp10 constructs.	12
**Figure S4**	Scattering profiles for nsp10 samples in SEC-SAXS	13
**Figure S5**	Dimensionless Kratky plots for the nsp10 proteins measured by SAXS	14

Table S1. Summary of nsp10 constructs from β-CoVs used in this study.

Protein construct and name	Residue numbering	Original polypetide numbering	Calculated MW after cleavage [Da]	Calculated pI after cleavage
**Short SARS-CoV-2 nsp10**	10-133	Asn4264 - Gln4385	13,272	7.70
**Long SARS-CoV-2 nsp10**	1-133	Ala4255 - Gln4385	14,026	6.70
**Full-length SARS-CoV-2 nsp10**	1-139	Ala4255 - Gln4391	15,022	6.70
**Full-length MERS nsp10**	1-140	Ala4238 - Gln4378	15,122	7.70
**Full-length SARS nsp10**	1-139	Ala4231-Gln4369	15,075	6.70

Table S2. SAXS data collection and scattering parameters for the β-CoV nsp10 proteins studied.

**Sample**	**SARS-CoV-2 nsp10**			**SARS nsp10**	**MERS nsp10**
	**full-length**	**long**	**short**	**full-length**	**full-length**
**Data Collection parameters**					
Instrument	B21 Beamline (Diamond Light Source, UK)				
Beam size at sample (mm^2)	1.0 × 0.25				
Wavelength (Å)	0.9464				
s-Range ($Å^{-1}$)	0.0045-0.3400				
Method	SEC-SAXS				
Sample to Detector D (mm)	3722.0				
Temperature (K)	288				
**Structural parameters**					
R_g (Å) (from Guinier)	17.10 (± 0.14)	16.35 (± 0.10)	15.20 (± 0.12)	17.19 (± 0.10)	17.27 (± 0.10)
R_g (Å) (from P(r))	17.31 (± 0.01)	16.64 (± 0.01)	15.46 (± 0.01)	17.40 (± 0.02)	17.32 (± 0.01)
D_{max} (Å)	59.0	57.0	51.4	59.4	53.3
Porod volume estimate ($Å^3$)*	26542	23162	20976	24726	24363
**Molecular mass determination**					
Molecular mass from Porod volume (Vp ∗ 0.6) (Da)	15925	13897	12585	14836	14618
Molecular mass from forward scattering (Da)	15395 (± 268)	13985 (± 291)	9324 (± 161)	20841 (± 63)	14328 (± 44)
Molecular mass from sequence (Da)	15151	14026	13272	14843	14891
**Software**					

Data processing	ATSAS v3.2.1 (CHROMIX, PRIMUS, GNOM)
Modelling	DAMMIF, DAMMIN (*ab initio*), BUNCH (rigid body)
Atomic models scattering	CRYSOL

*Porod Volume was calculated through Dammif.

A

Full-length SARS-CoV-2 nsp10

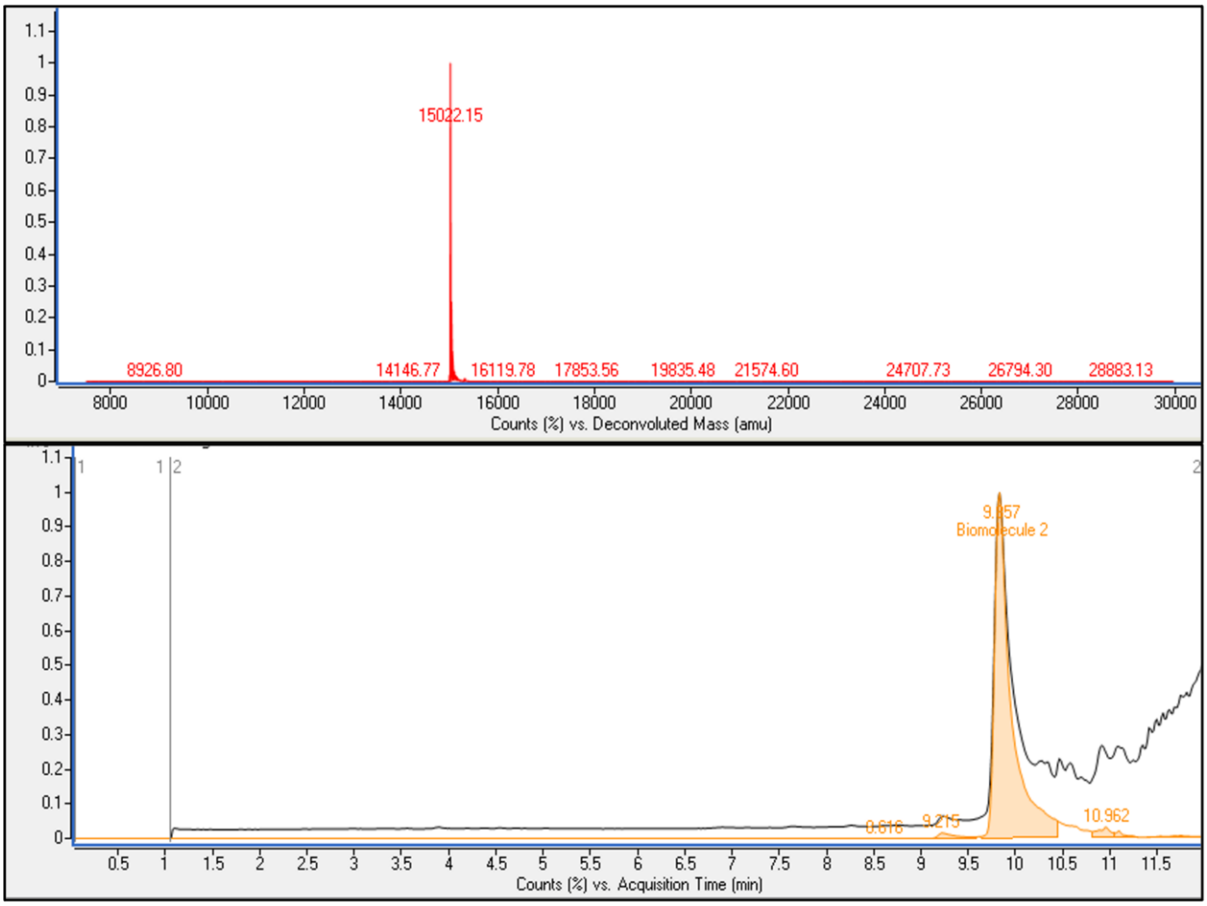

B

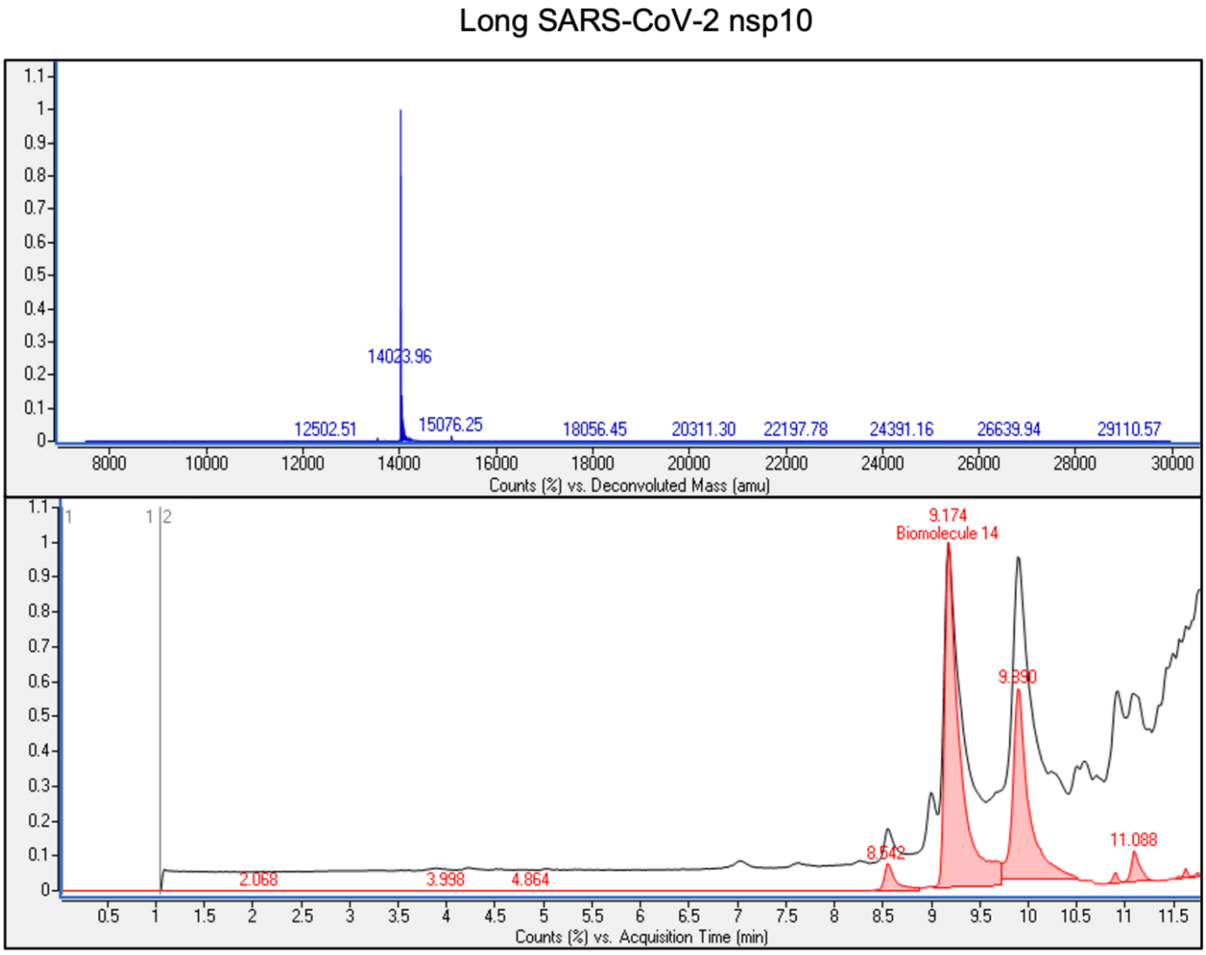

Long SARS-CoV-2 nsp10
14023.96
12502.51
15076.25
18056.45
20311.30
22197.78
24391.16
26639.94
29110.57
Counts (%) vs. Deconvoluted Mass (amu)
9.174
Biomolecule 14
9.890
8.542
11.088
2.068
3.998
4.864
Counts (%) vs. Acquisition Time (min)

C

Short SARS-CoV-2 nsp10

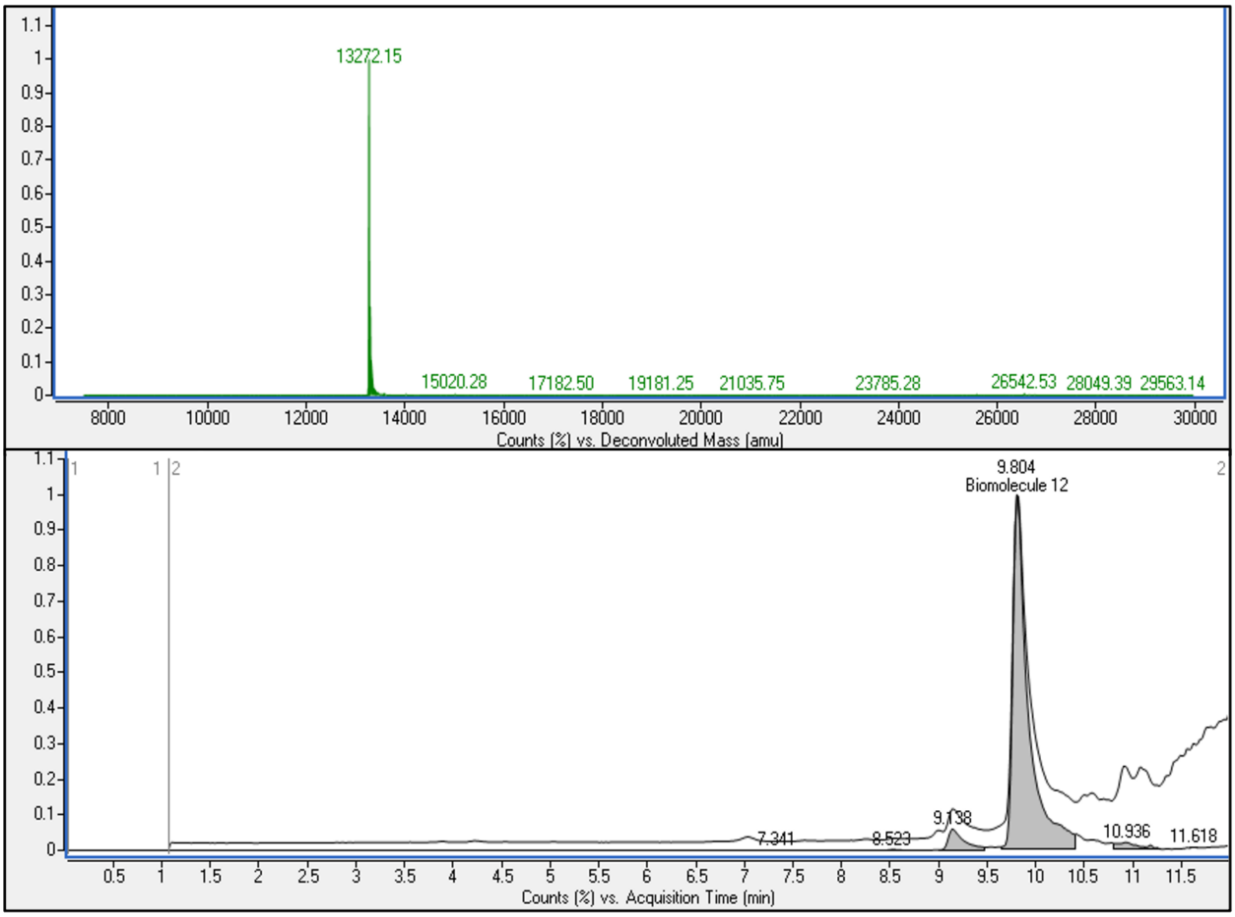

D

Full-length SARS nsp10

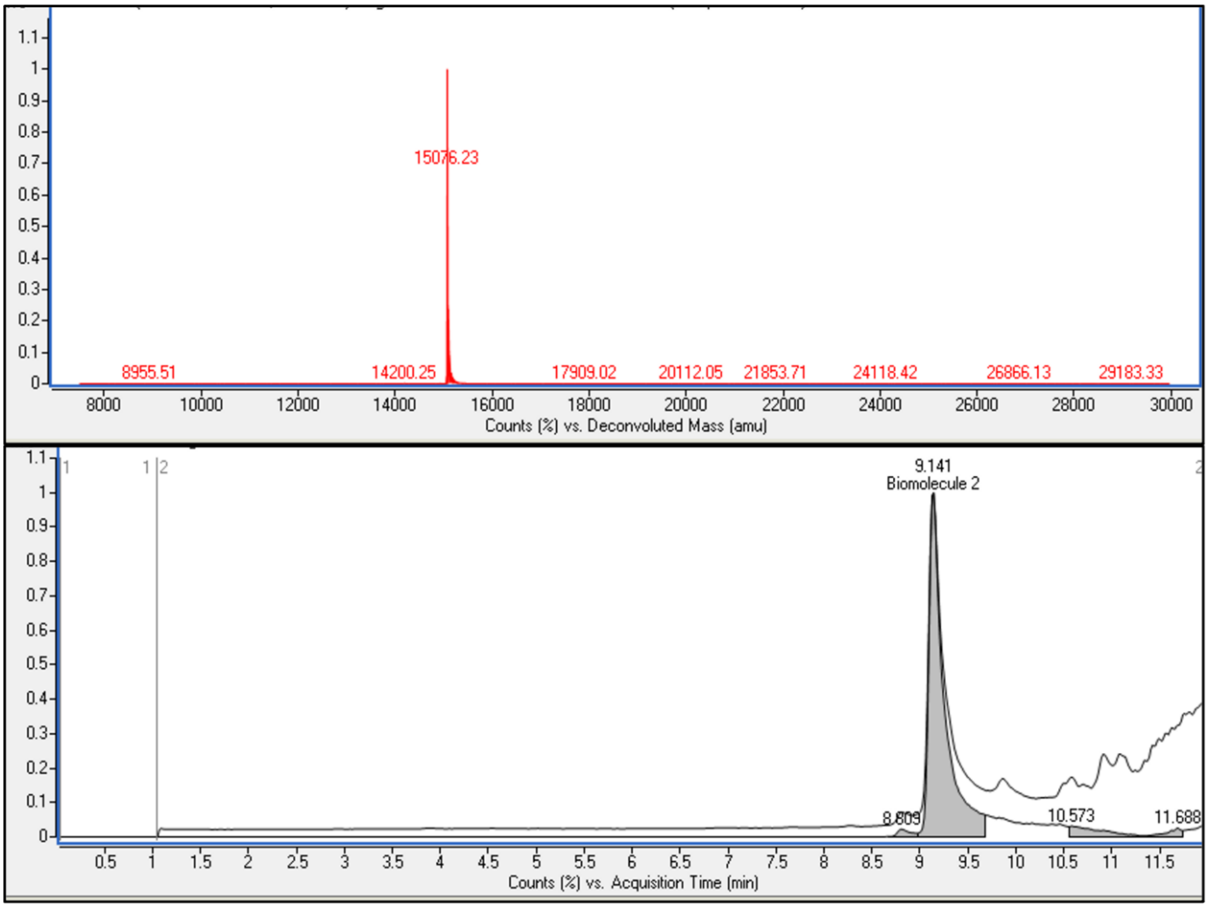

E

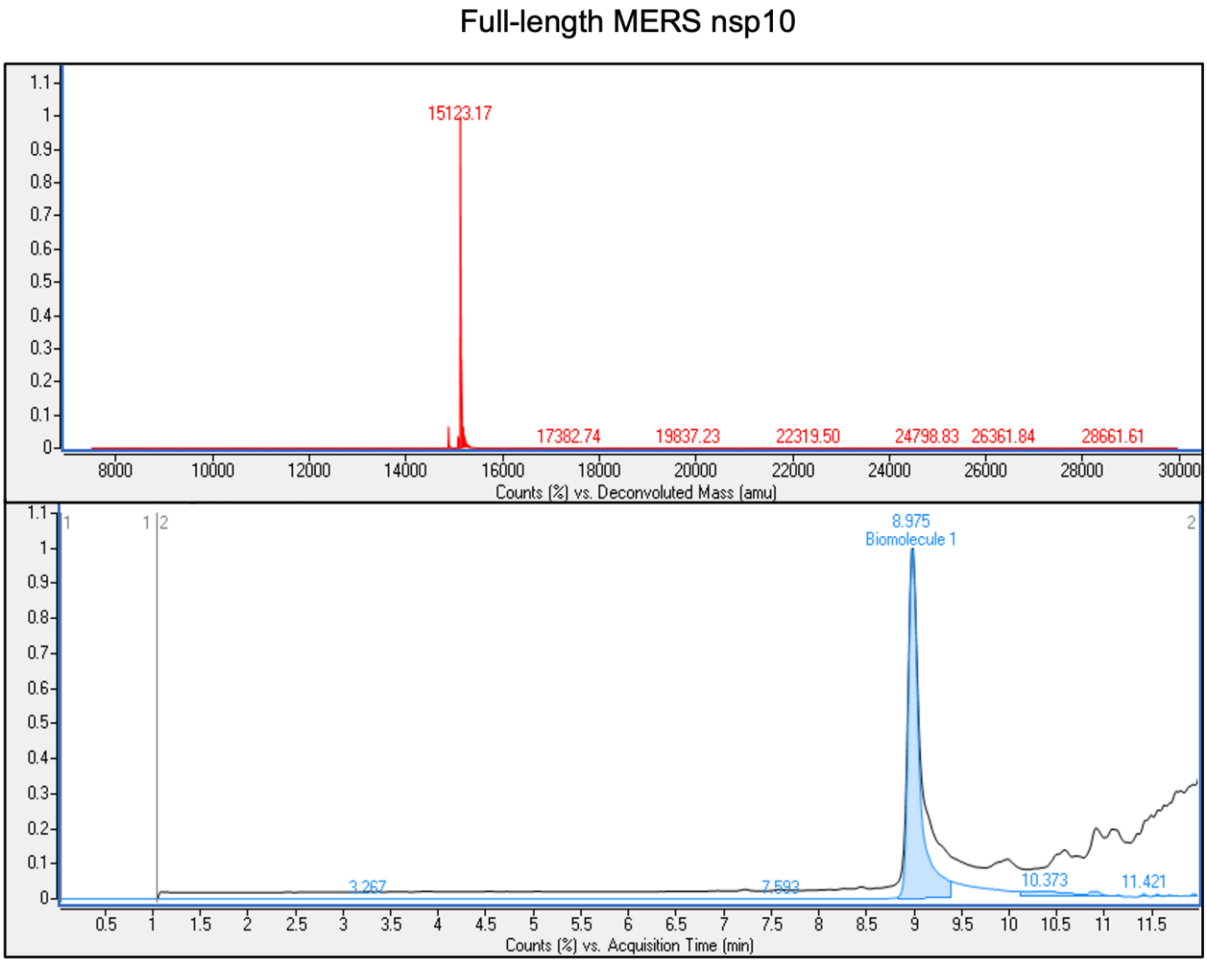

Figure S1. Denatured High-resolution mass spectra (HRMS) of **A)** full-length SARS-CoV-2 nsp10, **B)** long SARS-CoV-2 nsp10, **C)** short SARS-CoV-2 nsp10, **D)** full-length SARS nsp10 and **E)** full-length MERS nsp10. Deconvoluted QTOF-MS spectrums of each sample are showing in the upper panels. The signal-counts of the main peaks were normalised to 1. The calculated mass values were labelled on the top of main peaks. Chromatograms of each sample using reversed-phase chromatography are in the lower panels. The signal-counts of the main peaks were normalised to 1. The acquisition time was labelled on the top of main peaks.

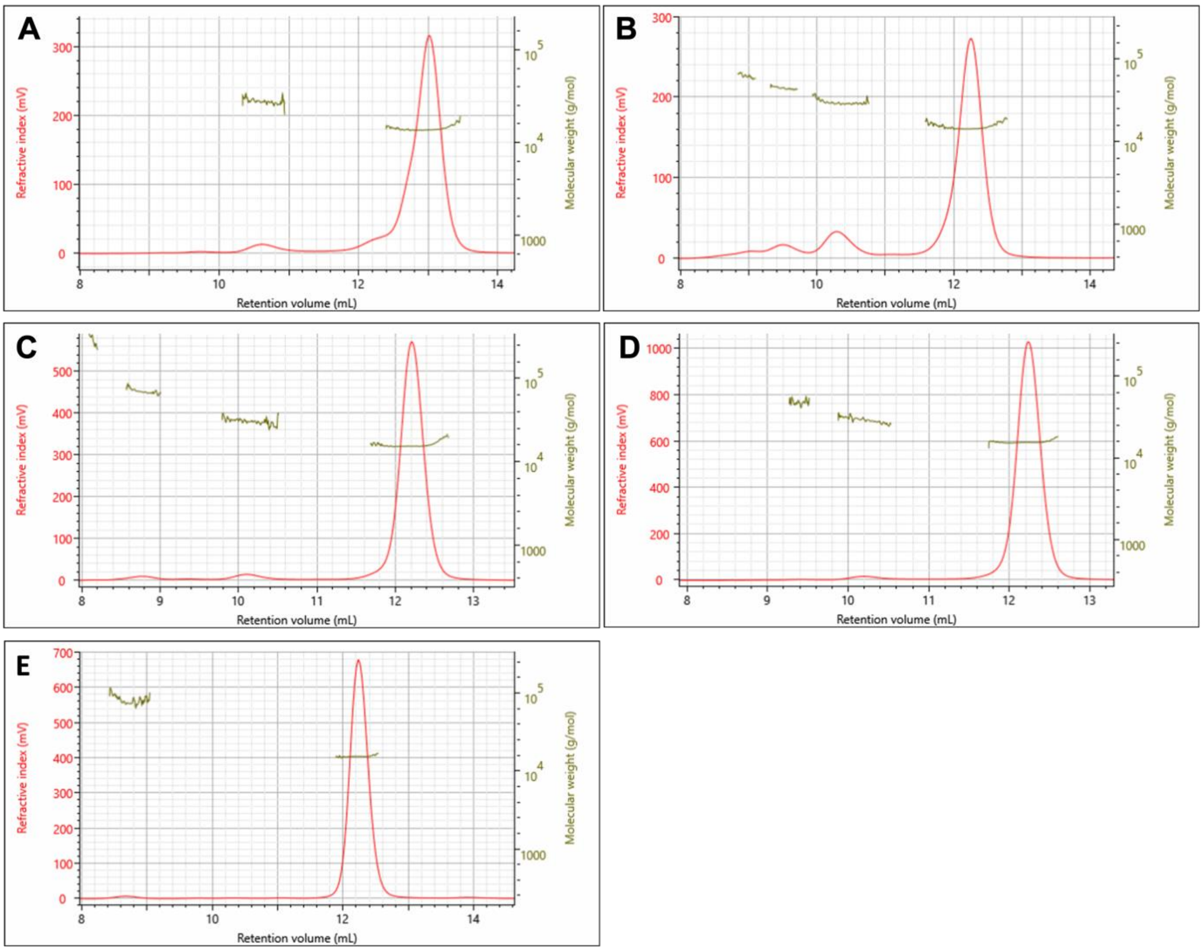

Figure S2. Representative OmniSEC traces. **A.** Short SARS-CoV-2 nsp10. **B.** Long SARS-CoV-2 nsp10. **C.** Full-length SARS-CoV-2 nsp10. **D.** Full-length SARS nsp10. **E.** Full-length MERS nsp10. The refractive indexes are shown in red; the determined molecular weight of the peaks are in green.

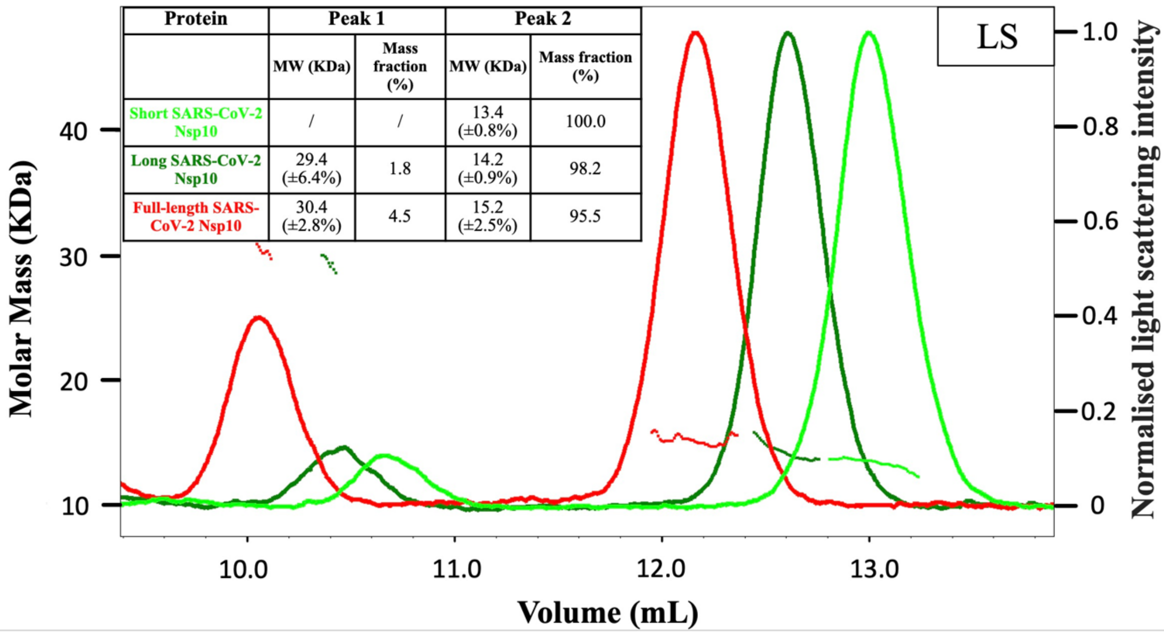

Protein	Peak 1		Peak 2	
	MW (KDa)	Mass fraction (%)	MW (KDa)	Mass fraction (%)
Short SARS-CoV-2 Nsp10	/	/	13.4 (±0.8%)	100.0
Long SARS-CoV-2 Nsp10	29.4 (±6.4%)	1.8	14.2 (±0.9%)	98.2
Full-length SARS-CoV-2 Nsp10	30.4 (±2.8%)	4.5	15.2 (±2.5%)	95.5

Figure S3. SEC-MALS analysis of three SARS-CoV-2 nsp10 constructs. Normalised light scattering (LS) peaks of short, long, and full-length SARS-CoV-2 nsp10s are coloured in light green, dark green, and red, respectively. The inset report on the molecular weight and mass fractions measured experimentally using SEC-MALS, where the lower peaks are categorised as peak 2 and the main peaks are named peak 2. The molecular weights for the second peak of short SARS-CoV-2 nsp10 was not statistically significant and therefore not included in the table.

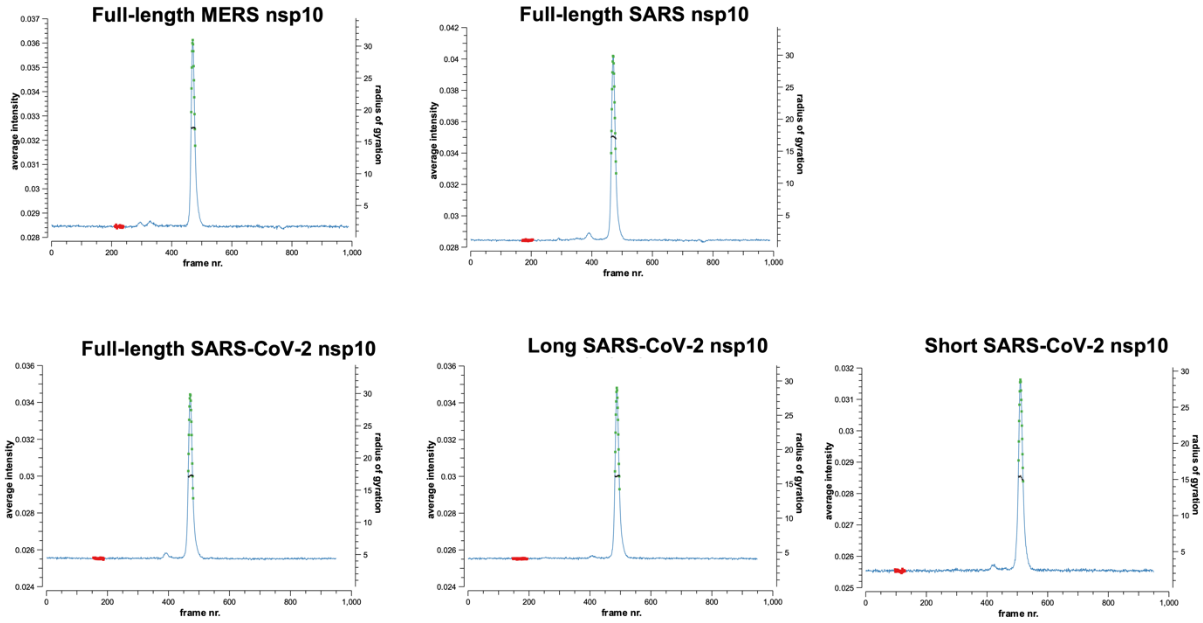

Figure S4. Scattering profiles for the nsp10 samples, measured by SEC/SAXS. The frames selected for buffer and peak calculations are shown as red dots. Peak frames were adjusted to generate a consistent R_g value (shown as black dots).

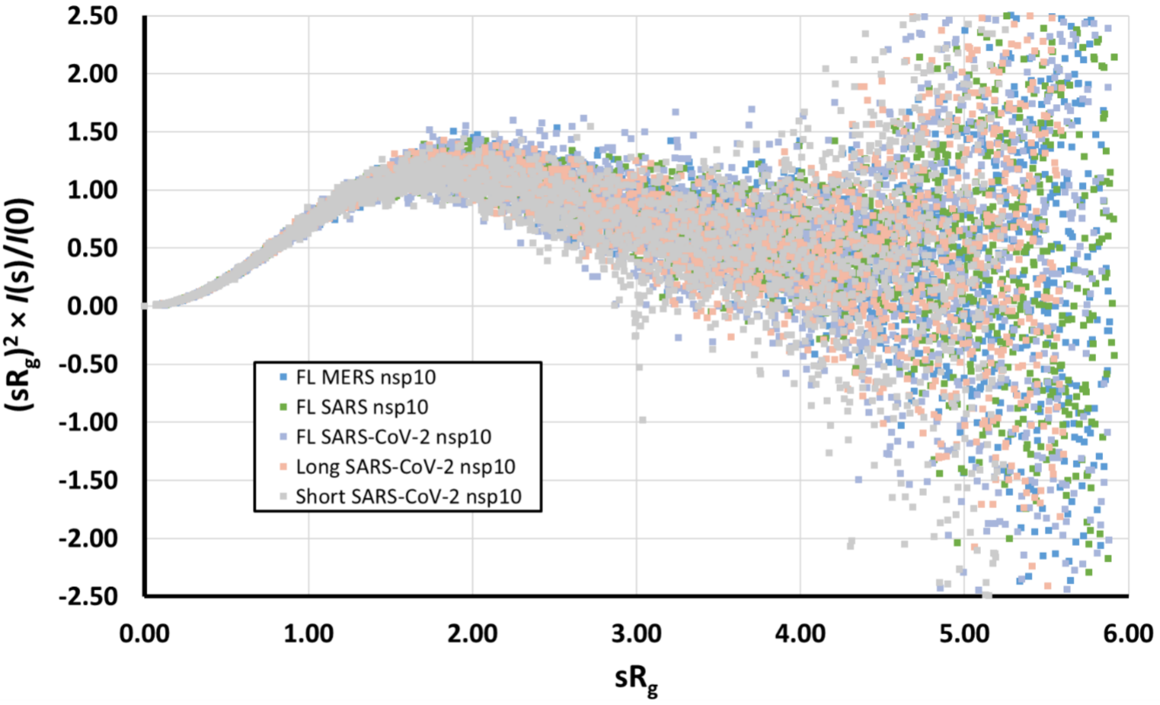

Figure S5. Dimensionless Kratky plots for the nsp10s measured by SAXS as shown in Fig 2.
